# Recent Advances in Leveraging AI for Scalable and Extensible Biomedical Knowledge Management

**DOI:** 10.1055/s-0046-1816628

**Published:** 2026-11-03

**Authors:** Gongbo Zhang, Betina Idnay, Chunhua Weng

**Affiliations:** 1 Columbia University, New York, NYUSA

**Keywords:** Knowledge Representation, Artificial Intelligence (AI), Symbolic AI, Scalability

## Abstract

**Objectives:** : To survey the recent literature on knowledge representation and management (KRM) and their biomedical applications.

**Methods** : A bibliometric analysis of KRM methodological innovations and health applications published from January 2024 to February 2025 was carried out.

**Results** : Four major methodological development trends emerge from our analysis, which are multimodal data integration, ontology-driven knowledge representation, continuous knowledge representation learning, and contrastive learning for knowledge extraction. These methods are applied across a range of biomedical domains, as represented by accelerated drug development, clinical decision support, and personalized medicine.

**Discussion** : There is a growing emphasis on data-driven and learning-based approaches to biomedical knowledge representation. However, challenges remain in developing standardized evaluation benchmarks and enabling continuous updates to knowledge resources. Future innovations may arise from integrating knowledge across overlapping domains—such as precision medicine and drug repurposing—to enhance the translational impact of KRM methods.

## 1. Introduction

Knowledge Representation and Management (KRM) is a cornerstone of medical informatics, enabling the systematic organization, storage, and retrieval of medical knowledge to support clinical decision support and biomedical research. Traditionally, KRM relied on rule-based systems and expert knowledge bases, which may organize complex medical concepts and their relationships using ontologies or concept-oriented terminologies, such as SNOMED CT, Medical Subject Headings (MeSH), Logical Observation Identifiers, Names, and Codes (LOINC), Human Phenotype Ontology (HPO), and Gene Ontology (GO), to ensure human comprehension and machine-readability. With the rise of artificial intelligence (AI) and machine learning (ML), KRM has evolved to incorporate data-driven approaches such as large-scale knowledge graphs, heterogeneous data integration, and natural language processing (NLP), exemplified by the recent integration of Unified Medical Language System (UMLS) with large language models (LLMs) to serve as a large knowledge graph [ 1 ]. These technologies enable real-time knowledge extraction and synthesis from heterogeneous biomedical data sources, including electronic health records (EHRs), clinical practice guidelines, and the scientific literature. As digital health continues to advance with telemedicine, remote monitoring, and personalized medicine, KRM must evolve to accommodate multimodal data, patient-generated information, and AI-driven insights, ensuring that healthcare providers have access to accurate, context-aware, and interpretable knowledge at the point of care.

Recent innovations in knowledge representation methodologies have focused on improving automated reasoning, enhancing biomedical inference, and integrating diverse data sources. Knowledge representation learning, particularly through knowledge graph embeddings [2–6], has been widely applied to encode structured and unstructured biomedical data, facilitating predictions for drug-disease interactions and gene-disease associations. Hybrid neural-symbolic reasoning approaches have gained traction by combining rule-based inference with deep learning models, allowing for more interpretable and context-aware decision-making in precision medicine [7–11]. Pretrained LLMs are increasingly integrated with biomedical knowledge graphs to support knowledge extraction, automated literature mining, and clinical text generation [12–17]. Additionally, contrastive learning techniques are being explored for drug discovery, disease phenotyping, and treatment optimization, refining entity relationships by distinguishing subtle biomedical similarities and differences [18–22]. Ontology-driven knowledge representation remains crucial for semantic interoperability, with recent priorities focused on aligning ontologies, expanding medical taxonomies, and enabling real-time updates to biomedical knowledge graphs [23–30].

These methodological advancements have translated into real-world applications across multiple healthcare domains. AI-augmented clinical decision support (CDS) systems leverage structured knowledge representations to integrate clinical guidelines, real-world evidence, and patient data to improve diagnosis, treatment recommendations, and predictive analytics [31–35]. In drug discovery, graph-based ML models have been used to accelerate drug repurposing, drug-target interaction predictions, and adverse event detection, addressing the challenges of data sparsity and complex biochemical interactions [36–45]. Disease prediction and risk assessment have benefited from multimodal data fusion, where patient-specific clinical, genomic, and imaging data are integrated into unified representations for early disease detection and personalized treatment strategies [46–54]. Advances in ontology-driven knowledge representation have enhanced precision medicine applications, enabling the stratification of disease subtypes, the identification of prognostic biomarkers, and the generation of AI-assisted clinical pathways [ 7 55 56 57 58 59 60 ]. The ability to construct and reason over multi-source biomedical knowledge graphs has also improved patient monitoring, facilitating real-time disease tracking, remote health assessments, and AI-driven intervention strategies [61–65]. As KRM continues to evolve, integrating generative AI, knowledge-driven inference, and multimodal biomedical data will play a key role in ensuring scalable, explainable, and AI-augmented healthcare systems.

This survey examined how AI has recently been leveraged in modern biomedical knowledge representation, discovery, and management. While this review may not be exhaustive, it synthesizes key methodological trends, real-world applications, and emerging challenges, outlining future directions for KRM in AI-augmented healthcare systems.

## 2. Methods

### 2.1 Search Strategy

We chose to focus on PubMed for our review given that it is the most comprehensive and authoritative source of peer-reviewed biomedical literature, aligning closely with our focus on biomedical KRM. While ACM and IEEE databases contain publications on generic ontology research methods, they cover fewer studies that directly address the biomedical and translational science topics at the core of our analysis. We used the following query to search the literature from PubMed published between January 1, 2024, and February 20, 2025, with a focus on KRM applications in healthcare:

**Table:** 

Query
“Knowledge Representation” OR “Knowledge Management” OR “Ontology” OR “Knowledge Graph” OR “Representation Learning” OR “Ontology Learning” OR “Taxonomy Learning” AND (“2024/01/01” [Date – Publication]: “2025/02/20” [Date – Publication])”

### 2.2 Screening and Selection of Literature

The initial search retrieved 7,231 publication records. We filtered out records that do not explicitly mention knowledge representation, knowledge graphs, and taxonomy learning in the title, abstract, keywords, or MeSH terms. After this step of filtering, 528 articles were retained. To further identify key topics, we first used a cutting-edge LLM, o3-mini [ 66 ], developed by OpenAI, to generate three to six key phrases characterizing each study. These key phrases were manually examined, leading to the identification of two broader categories: methodologies and applications. To ensure a healthcare-centered focus, we excluded studies that solely contributed to theoretical frameworks or computational models without demonstrating tangible healthcare applications. After this filtering step, 327 relevant studies were retained. To streamline the analysis, we manually selected a subsample of 83 articles from the 327 publications that specifically highlight the use of generative AI methods for knowledge management. This subsample was chosen to ensure a balanced representation across the identified topics (see details in Section 3) based on manual assessment. While this approach may limit the overall comprehensiveness, it enables a more focused exploration of topic-level trends within the literature.

### 2.3 Data Extraction

A spreadsheet was created by the first author and the last author to tag an initial sample of 41 articles. Based on the preliminary labels from this sample, the first author developed a structured table to extract information related to key methodological innovations in KRM, as well as prominent application scenarios. The extracted information was then independently reviewed by the second author for accuracy and completeness. Any discrepancies were resolved through discussion, with input from a third author when necessary.

## 3. Results

Within the past year, notable progress has been observed in leveraging AI technologies to improve the diversity of the data sources for knowledge representation through multimodal data integration, improving the extensibility of knowledge representations via ontology-driven knowledge representation, and automating knowledge representation learning ( Figure 2 ). Popular applications include: (a) accelerated drug development, (b) clinical decision support, (c) personalized medicine, and (d) disease prediction and risk assessment. We note that these application domains are not mutually exclusive, as many studies overlap across multiple methodological approaches and applications, reflecting the interdisciplinary nature of KRM in healthcare. Supplementary Table 1 shows the main advances in knowledge representation within the past year.

**Figure 1.**
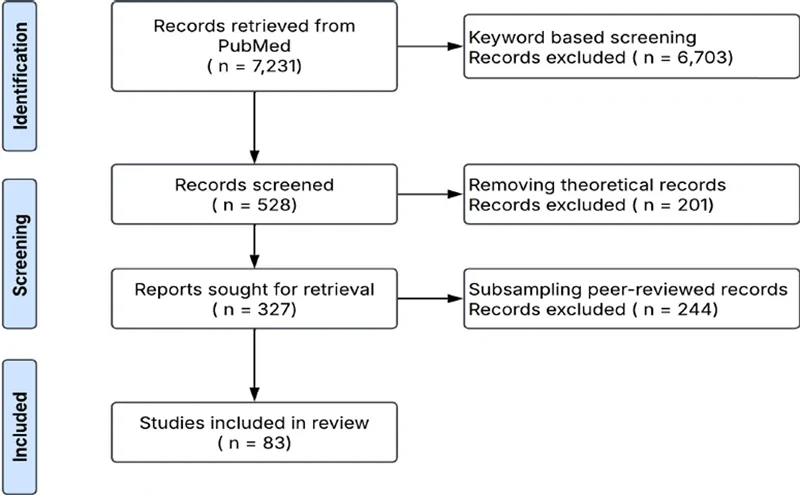
Search strategy and selection process for this KRM survey.

**Figure 2.**
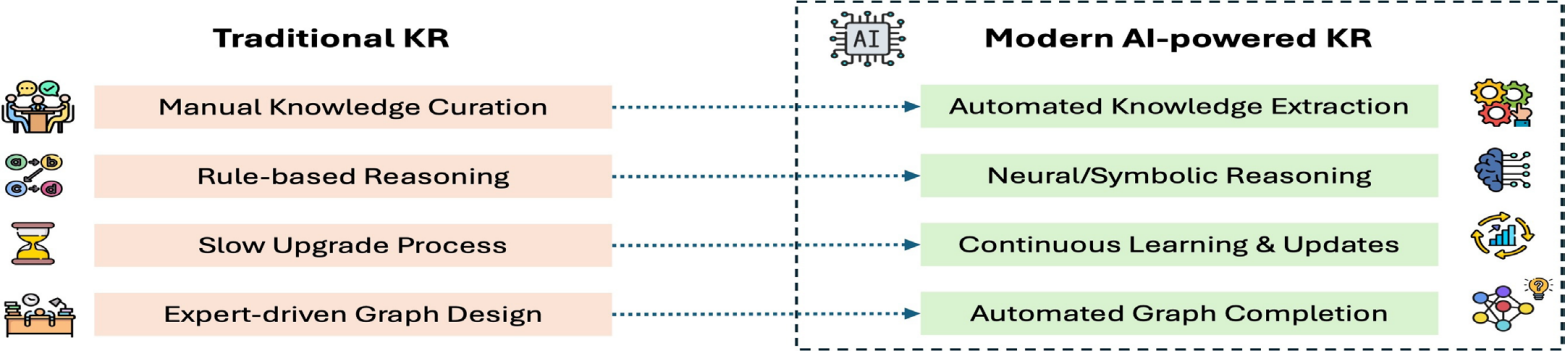
AI-powered knowledge representation.

### 3.1 Methodology Advances

#### 3.1.1 Multimodal Data Integration

Multimodal data integration refers to the combination and alignment of heterogeneous biomedical data sources, including clinical text, medical images, and genomics, into a unified knowledge representation. This integration enables ML models to capture complex relationships across diverse biomedical domains, facilitating more comprehensive insights into health and disease. One key area of research in this field is heterogeneous data fusion. Podkolodnyy *et al.* explored the integration of heterogeneous experimental data in bioinformatics, systems biology, and biomedicine [ 67 ]. Blobel *et al.* advanced interoperability in healthcare ecosystems by integrating genomic data, EHRs, and medical imaging data, ensuring seamless data exchange between health and social care systems [ 68 ]. To enhance biomedical knowledge graph interoperability, Mohseni Ahooyi *et al.* integrated human genomic data across scales ranging from base pairs to entire chromosomes originating from different genome builds [ 69 ]. To improve the prediction of microbe-drug associations, Gu *et al.* proposed a predictive model based on a knowledge graph integrating microbe, disease, and drug entities [ 70 ]. Diamant *et al.* introduced an upgrade to the Harmonizome database, which involved 26 information sources of associations between genes and various attribute types, such as cells and pathways [ 71 ].

Another approach is encoding different data types into a shared latent space, which preserves modality-specific information. Luo *et al.* proposed a drug repurposing model that encodes biomedical knowledge graph, drug, and disease similarity data [ 2 ]. To support drug-target affinity prediction, other drug-related studies developed a shared representation space for molecular structure and protein sequence and similarity among drugs and proteins [ 72 73 ]. To facilitate drug-gene/protein interaction discovery, Chang *et al.* merged LLM-based text embeddings with knowledge graph embeddings to represent biomedical entities [ 74 ]. Li *et al.* employed dual generative adversarial networks to align molecular structure and knowledge graph embeddings to support drug-drug interactions [ 75 ]. Theodoropoulos *et al.* developed a knowledge graph, which encodes both structured data like vital signs and medication lists and unstructured data, including physician notes, radiology and pathology reports, to support a predictive model for hospital readmission [ 76 ].

A group of studies focused on cross-modal reasoning, where the knowledge graphs were constructed to link diverse biomedical data, enabling AI systems to infer relationships between entities such as genes, drugs, and diseases. Li *et al.* approached circular RNA (circRNA)-disease association (CDA) as predicting missing links on a multimodal knowledge graph that contains existing CDA, gene expression, and regulation information [ 77 ]. Firoozbakht *et al.* developed a knowledge graph representation for inferring molecular mechanisms for adverse drug reactions (ADRs), where the knowledge graph integrated information about drug-ADR associations, disease-phenotype relationships, and protein-protein interactions [ 78 ]. A few studies integrated patient-specific data and biomedical knowledge graphs to support precision medicine [ 79 80 ] and rare disease diagnosis [ 81 ].

#### 3.1.2 Ontology-Driven Knowledge Representation

Besides integrating multimodal data, progress has been made in integrating knowledge from diverse biomedical ontologies for large-scale knowledge integration [ 82 ]. Ontologies formally represent knowledge in a domain, defining entities, attributes, and relationships. A recent trend focuses on ontology alignment and expansion, which harmonizes multiple ontologies to create an interoperable knowledge graph and dynamically updates knowledge graphs as new biomedical knowledge emerges. Ontology-driven knowledge representation involves building structured representations by harmonizing domain-specific ontologies with large-scale data sources. Wu *et al.* proposed a zero-shot ontology extension framework that dynamically updates an existing symptom taxonomy among breast and bladder cancer survivors using online and hierarchical clustering to incorporate new concepts, such as symptoms identified from patient forum data [ 23 ]. Toro *et al.* introduced DRAGON-AI, which generates logical ontology components by extraction from multiple existing ontologies and textual data sources [ 24 ]. Cui *et al.* discussed efforts to align multiple ontologies and dynamically update knowledge graphs by incorporating new biomedical knowledge, such as identifying missing concepts in SNOMED CT and developing a COVID-19 interface terminology for automatic EHR annotations [ 25 ]. Dally *et al.* discussed the development of an ontology that models the social determinants of health (SDoH), which aligns SDoH concepts with biomedical ontologies through semantic links, incorporating new concepts such as socioeconomic status, work conditions, and psychosocial factors [ 26 ]. Chatterjee *et al.* constructed a knowledge graph with the SWELL-KW dataset, enhanced by a domain ontology to improve data representation and knowledge acquisition for heart rate variability analysis, particularly in stress level classification [ 27 ]. Cao *et al.* used Human Phenotype and Orphanet ontologies to build a knowledge graph to automate the extraction of rare disease-related information, connecting diseases to their associated clinical features and other medical concepts [ 28 ]. Mazein *et al.* presented MeDaX, a knowledge graph constructed from synthetic patient data that utilizes a generalized extract-transform-load process for ontological structuring, focusing on converting Fast Healthcare Interoperability Resources (FHIR) data into a graph format for clinical research [ 29 ]. Vogt *et al.* discussed using semantic units to structure a knowledge graph, where semantic units are aligned with different ontological and discursive information [ 30 ].

In addition to ontology alignment and expansion, other studies also investigated the integration of ontology with unstructured information. Homo Sapiens Chromosomal Location Ontology was designed and developed to facilitate the integration and analysis of human genomic data across scales from the whole chromosomes to individual base pairs [ 69 ]. Wang *et al.* developed a knowledge network platform, CancerPro, for examining pan-cancer prognostic markers and investigating genes or drugs of interest, which integrates comprehensive biomedical data from ontology and knowledge graphs [ 83 ]. Altuhaifa *et al.* aggregated predefined terms from existing ontologies, including the Human Disease Ontology, Symptom Ontology, and Human Developmental Anatomy Ontology, to develop an ontology for fall risk management [ 84 ]. Yu *et al.* constructed a prostate cancer (PCa) ontology, aggregating PCa-associated genes and lifestyle data to support precision diagnosis and treatment [ 85 ]. Gao *et al.* built a taxonomy upon existing terms and models for quality improvement initiatives focusing on employee wellness in healthcare organizations [ 86 ].

#### 3.1.3 Continuous Knowledge Representation Learning

Conventional knowledge representations are often static and entail formidable costs to be updated or extended. The latest AI technologies have enabled Knowledge Representation Learning (KRL), which focuses on encoding structured and unstructured knowledge into continuous vector spaces, allowing machines to understand, reason, and infer new facts based on existing knowledge. A key advancement in KRL is the use of knowledge graph embeddings, which capture the relationships between biomedical entities and improve downstream predictive tasks. Luo *et al.* represented drug and disease entities as topological feature embeddings within biomedical knowledge graphs, enabling more accurate predictions of drug-disease interactions [ 2 ]. Li *et al.* explored a different approach by modeling biomedical literature as knowledge graphs, demonstrating that this method effectively captures gene pathogenicity information, facilitating large-scale gene-disease relationship analysis and understanding [ 3 ]. To support the treatment of antibiotic-resistant bacterial infections, Pan *et al.* constructed knowledge graphs to map associations between bacteriophages and their hosts, providing valuable insights for therapeutic interventions [ 4 ]. Gan *et al.* proposed an algorithm that generates representations from both codified and narrative features, which has been successfully applied to predictive modeling tasks, including disease phenotyping and drug side effect prediction [ 5 ]. Zhu *et al.* introduced a novel neighbor-distance model for entity alignment across multiple knowledge graphs, improving the integration and interoperability of biomedical knowledge sources [ 6 ].

Neural-symbolic representation, a hybrid knowledge representation approach that combines data-driven learning with knowledge-driven reasoning, has gained increasing attention in the research community. Yang *et al.* developed a precision medicine knowledge base that integrates drug, gene, and disease information into knowledge graphs and provides textual explanations of subgraphs using LLMs [ 7 ]. Jiménez *et al.* proposed an architecture for drug repurposing that represents compounds, diseases, and genes in knowledge graphs and predicts potential drug-disease relationships using a neural predictive module [ 8 ]. A computer-aided diagnosis system for thyroid nodules was proposed based on a multi-grained domain knowledge representation and neural networks for predicting thyroid nodule malignancy [ 9 ]. Sudhahar *et al.* explained drug repurposing predictions using a hybrid approach that generates rules and paths explaining drug effects on diseases with knowledge graphs and improves path selection with a reinforcement learning knowledge graph completion model [ 10 ]. Yue *et al.* proposed a bladder tumor segmentation method where clinical knowledge is represented as rules in convolutional neural networks [ 11 ].

Pre-trained LLMs have demonstrated great potential for various biomedical text mining tasks [ 66 87 – 97 ] and have been leveraged for biomedical knowledge representation. Manicai *et al.* evaluated LLMs for producing concept maps from otolaryngology guidelines [ 12 ]. Yang *et al.* examined LongFormer, a pre-trained LLM, and disease co-occurrence maps to improve medical knowledge representation for automated International Classification of Diseases (ICD) code classification [ 13 ]. Ivanisenko *et al.* leveraged LLMs to enhance protein interaction predictions and identified 25 interactions absent in existing knowledge bases [ 14 ]. Azam *et al.* argued that LLMs can be mined as biological knowledge graphs and surveyed 21 LLMs on gene regulatory relation prediction [ 15 ]. Xiao *et al.* leveraged LLM's pre-trained text embeddings and knowledge graphs to improve link predictions on biomedical knowledge graphs [ 16 ]. Fang *et al.* used GPT-4 to construct a drug indication taxonomy from free-text drug labels [ 17 ].

#### 3.1.4 Contrastive Learning for Knowledge Extraction

Contrastive learning, a self-supervised learning technique for distinguishing between similar and dissimilar pairs of entities, has been employed to infer relationships among entities. Gong *et al.* introduced a contrastive loss that captures similarities and differences between multiple molecules to incorporate chemical knowledge into their multipath information fused self-supervised framework for drug discovery [ 18 ]. Xie *et al.* formulated chemical reactions as knowledge graphs, which used a contrastive loss function that brings the embeddings of reactants and products closer in the vector space while distancing unrelated entities [ 19 ]. Lian *et al.* developed a chest X-ray report generation system that employs contrastive learning to bridge the semantic gap between chest X-ray images and medical reports [ 20 ]. Zhong *et al.* developed a knowledge graph-based pipeline for drug-drug interaction (DDI) prediction, incorporating a contrastive learning module that utilizes horizontal reversal to generate high-quality embeddings for drug pairs [ 21 ]. Gao *et al.* used multi-modal knowledge graphs with contrastive graph attention to capture nuanced similarities among ophthalmology medical records [ 22 ].

### 3.2 Primary Biomedical Applications

#### 3.2.1 Accelerated Drug Development

Novel representation techniques have been employed to accelerate drug discovery via uncovering novel drug-drug interactions (DDIs) or facilitating drug repurposing. Luo *et al.* reviewed the integration of deep learning with knowledge graphs to predict DDIs, addressing the challenges of data sparsity and complexity in existing DDI prediction models [ 36 ]. Lin *et al.* investigated feature fusion of information from multiple data sources [ 37 ]. Zhong *et al.* explored contrastive learning to obtain better drug representation using horizontal reversal techniques [ 21 ]. Biomedical knowledge graphs have also played a pivotal role in accelerating drug repurposing, where patient, drug, and disease information were integrated to predict novel treatment strategies [ 2 16 38 78 80 98 ]. Kim *et al.* introduced a neural network model, ExPDrug, to interpret different pathways and prioritize drug candidates [ 40 ]. Various tools, such as TarKG, TTModel, and TarIKGC, have been developed for target prioritization with a heterogeneous drug-target-disease network [41–44]. Selote *et al.* created a knowledge graph to propose drug candidates for Alzheimer's, Parkinson's, and glioma disease [ 99 ]. Huang *et al.* introduced TxGNN, a graph foundation model for zero-shot drug repurposing [ 45 ].

#### 3.2.2 Clinical Decision Support

Recent advancements in knowledge graphs, AI models, and real-time data integration have also been applied to support clinical decision-making. Ebben *et al.* integrated rule-based decision logic with real-world data, enhancing the transparency and interpretability of clinical decision-making processes [ 31 ]. By translating narrative guidelines into clinical decision trees using information standards, the study improves the accessibility and effectiveness of decision support tools, as exemplified by the digitization of the Dutch breast cancer guideline. Afshar *et al.* grounded the LLM diagnosis predictions by providing the UMLS knowledge and demonstrated the benefit of augmentation [ 32 ]. The study by Shang *et al.* proposed a knowledge graph-based system that integrates fragmented EHR data across multiple hospitals to enable collaborative reasoning and provide comprehensive, privacy-preserving decision support for diagnosing conditions like chronic kidney disease [ 33 ]. Li *et al.* developed a Gastric Cancer Clinical Decision Support System (GC-CDSS) built on a knowledge graph derived from Chinese treatment guidelines aimed at improving treatment decision-making by providing dynamic, personalized recommendations [ 34 ]. By integrating graph embedding algorithms like Node2Vec and GraphSAGE, GC-CDSS was evaluated through a retrospective cohort study, showing a 92.96% consistency with multidisciplinary team treatment decisions and demonstrating its potential to assist oncologists and promote standardized care in primary healthcare settings. Wang *et al.* introduced MDPG, a diagnosis prediction model based on patient knowledge graphs, which captures spatial, temporal, and spatial-temporal correlations in EHRs to enhance the prediction of patients' future states through multi-label classification [ 35 ].

#### 3.2.3 Personalized Medicine

Recent studies have increasingly applied knowledge graphs to construct patient-specific models that enable personalized treatment planning [ 55 ]. Mirthica *et al.* developed a safe drug recommendation system that leverages a knowledge graph to predict DDIs and provide personalized drug recommendations based on integrated patient, disease, and drug data [ 56 ]. Computational models, such as digital twins, were combined with personal health knowledge graphs (PHKGs) to provide real-time, patient-centric care [ 57 58 ], where the PHKGs were designed to dynamically incorporate new knowledge from the patients. Yang *et al.* developed CPMKG, a condition-based knowledge platform that integrates genetic-specific drug information into a comprehensive knowledge graph, enabling personalized medicine by facilitating drug-centric exploration, multi-knowledge inference, and LLM-powered textual interpretations [ 7 ]. Liu *et al.* developed DrugFormer, a graph-augmented LLM that integrates serialized gene tokens and gene-based knowledge graphs to predict drug resistance at the single-cell level tailored for individual patients [ 59 ]. Kakalou *et al.* developed the HEALIE knowledge graph by integrating medical ontologies and expert insights to model cognitive and socioeconomic factors and combined it with LLMs to generate personalized medical content [ 60 ].

In addition to treatment personalization, knowledge graphs are increasingly utilized to optimize treatment strategies and improve patient monitoring. Kunes *et al.* introduced Spectra for single-cell gene expression analysis to support refining immunotherapy strategies [ 61 ]. Xu *et al.* designed a framework for optimizing the control of a gait rehabilitation knee exoskeleton [ 62 ]. Blanchard *et al.* utilized knowledge graphs and network analysis to categorize and structure mobile health content for fibromyalgia [ 63 ]. Moens *et al.* built a context-aware visualization recommender system, VisCARS, leveraging knowledge graphs and collaborative filtering to personalize dashboards, enhancing patient monitoring in smart healthcare applications [ 64 ]. Li *et al.* developed a smart care system for the elderly using a dynamic knowledge graph that integrates personal, medical, and nursing data to provide personalized care recommendations [ 65 ].

Recent advancements in knowledge representation and graph-based modeling have also shown promise in improving prognostic accuracy, uncovering novel disease associations, and supporting early detection across diverse medical conditions. Shi *et al.* employed a knowledge graph-based semantic query method that identified a significant correlation between anxiety and non-suicidal self-injury, which highlights the need for further research to establish causality [ 46 ]. Xing *et al.* introduced a multi-omics graph knowledge representation model that integrates computed tomography imaging and non-imaging omics data with a graph convolutional network to improve early prognostic prediction for pneumonia patients [ 47 ]. Zeng *et al.* proposed KMGCN, a multi-graph neural network that integrates individual- and population-level features using both knowledge-driven and data-driven approaches for brain network analysis [ 48 ]. Huang *et al.* developed a web-based agent leveraging knowledge graph technology to monitor “tree hole” social media posts for suicidal signals and facilitate real-time suicide intervention, resulting in over 6,600 prevented suicides from 2018 to 2023 [ 49 ]. Wang *et al.* introduced KGRACDA, to improve circRNA-disease association prediction by integrating recursive subgraph construction and attention aggregation [ 50 ]. Ye *et al.* constructed a knowledge graph of statistically significant risk factors for Alzheimer's disease and age-related macular degeneration and conducted an environment-wide association study using UK Biobank data to identify shared environmental factors, ultimately generating a shared etiology landscape for both diseases [ 51 ]. Valencia-Moreno *et al.* proposed a breast cancer risk estimation model for Cuban women that integrates knowledge representation and ML algorithms, demonstrating improved prediction accuracy compared to models trained on US Hispanic populations [ 52 ]. Gualdi *et al.* constructed a biomedical knowledge graph and developed two novel embedding algorithms, Dlemb and BioKG2vec, demonstrating that knowledge graph embeddings can effectively predict disease-associated genes [ 53 ]. Lee *et al.* proposed a knowledge graph neural network that predicts recurrence risk for hormone receptor-positive breast cancer patients while elucidating regulatory pathways of multi-gene assays [ 54 ].

## 4. Discussion

Over the past two decades, the expansion of “Big Data in Medicine” due to the burgeoning development of biobanks, EHR data, patient-generated data, genomic data, and social determinants of health data has undeniably given rise to “Big Knowledge for Precision Health”. Biomedical knowledge representation is now increasingly driven by diverse, large-scale multimodal data and advancements in technologies that harmonize and merge multiscale biomedical knowledge across disparate ontologies. This evolution has transitioned the field from manual knowledge representation, constrained by the efficiency of knowledge engineers or domain experts, to high-throughput, scalable, and automated knowledge graph construction and KRL derived dynamically from big data. These innovations facilitate better integration of data with knowledge, enabling further discoveries and applications and bringing us closer to the vision of “Building biomedical knowledge networks to drive Precision Medicine” [ 100 ]. Notably, the past year has witnessed the rapid adoption of LLMs for ontology learning, alignment and harmonization, knowledge distillation, and knowledge graph construction, leading to more seamless integration between “Big Data” and “Big Knowledge”. These technological breakthroughs overcome previous knowledge representation bottlenecks, ensuring that the resulting knowledge networks are scalable, extensible, and remain current with the latest information.

We identified three broad areas of challenges and future opportunities. First, despite the abundance of application studies on biomedical knowledge graphs, there is a lack of standardized benchmarks for systematic comparisons among different approaches. As exemplified by the studies on drug repurposing, some studies, such as TarKG, TTModel, and TarIKGC, developed tools based on heterogeneous drug-target-disease networks [ 41 42 44 ], while others focused on neural networks or foundation models [ 40 43 45 ]. Even though these methods share similar objectives, their evaluations rely on different datasets, metrics, and experimental setups, making direct comparisons challenging. To advance KRM in biomedicine, it is crucial to establish widely accepted, standardized benchmarks that define common datasets, tasks, and evaluation metrics, ensuring fair comparisons and fostering reproducibility in future research.

Second, constructing comprehensive and reliable knowledge graphs requires continuous learning and automated updates. Current studies have explored integrating multiple sources of knowledge, for example combining biomedical literature, clinical trial data, and entity association networks into unified knowledge graphs [67–69,71,74,76]. However, these studies do not explicitly discuss how to continuously keep pace with newly published research, updated clinical guidelines, or emerging drug interactions in the included sources. The lack of automated knowledge updates limits the timeliness and reliability of knowledge graphs for real-world applications. Future efforts should focus on developing self-updating mechanisms, such as incremental learning approaches, reinforcement learning-based knowledge refinement, and real-time data ingestion pipelines, to ensure that knowledge graphs remain accurate, comprehensive, and updated.

Third, future research opportunities can emerge from distinct yet overlapping domains. For example, precision medicine and drug repurposing are traditionally considered separate fields—precision medicine focuses on tailoring treatments based on an individual's genetic and molecular profile [ 46 49 57 59 ], while drug repurposing identifies new therapeutic uses for existing drugs at a broader population level [40–43,45]. The intersection of these domains, known as precision drug repurposing, leverages biomedical knowledge graphs, patient stratification techniques, and AI-driven drug-disease association models to identify repurposed drugs tailored to specific patient subgroups [ 80 ]. This convergence not only enhances treatment efficacy but also accelerates the discovery of personalized therapeutic interventions. As knowledge graphs continue to evolve, integrating methodologies from overlapping fields will open new directions for targeted drug discovery and individualized treatment strategies, ultimately improving patient outcomes.

This study has several limitations. Our search was restricted to the PubMed database, which primarily covers biomedical and life sciences literature. Consequently, we may have missed relevant publications from computer science and AI more broadly, which are often indexed in databases like DBLP, IEEE Xplore, ACM Digital Library, and ACL Anthology. However, this focus aligns with our primary interest in biomedical knowledge management and its specific challenges. We intentionally prioritized studies directly addressing biomedical KRM over more general AI advancements, which may have led to the exclusion of foundational methodological work without direct biomedical applications. Future research could broaden this scope to include a wider range of technical literature, offering a more comprehensive perspective on KRM innovations across disciplines.

## 5. Conclusion

This work surveyed recent advancements in KRM, with a focus on methodological innovation and their healthcare applications. We observed a growing emphasis on multimodal data integration, ontology-driven knowledge graph construction, continuous learning of knowledge representations, and the use of contrastive learning techniques for knowledge extraction. These innovations are driving impactful applications in areas such as accelerated drug development, clinical decision support, and personalized medicine. Despite notable progress, several challenges and opportunities remain. Future research may benefit from the development of standardized benchmarks for evaluation, methods for continuous updating and refinement of knowledge graphs, and strategies for integrating knowledge across overlapping domains such as precision medicine and drug repurposing. Advancing KRM in these directions will be critical to supporting more dynamic, scalable, and clinically meaningful biomedical knowledge systems.
